# Multi-omics profiling of pancreatic neuroendocrine tumors: Interplay of genomic instability and the tumor microenvironment (Review)

**DOI:** 10.3892/ol.2026.15630

**Published:** 2026-04-28

**Authors:** Yin Wan, Yingnan Zhang, Philip A. Philip, Herbert Chen, Bassel El-Rayes, Asfar S. Azmi, Yang Shi

**Affiliations:** 1 Karmanos Cancer Institute, Department of Oncology, Wayne State University School of Medicine, Detroit, MI 48201, USA; 2 Department of Pharmacology, Wayne State University School of Medicine, Detroit, MI 48201, USA; 3 Henry Ford Health System, Detroit, MI 48202, USA; 4 Department of Surgery, University of Alabama at Birmingham, Birmingham, AL 35294, USA; 5 Division of Hematology and Oncology, Department of Medicine, O'Neal Comprehensive Cancer Center, University of Alabama at Birmingham Heersink School of Medicine, Birmingham, AL 35233, USA

**Keywords:** pancreatic neuroendocrine tumors, multi-omics profiling, tumor microenvironment, genomic instability, immune modulation, stromal remodeling

## Abstract

Pancreatic neuroendocrine tumors (PNETs) represent a biologically heterogeneous group of neoplasms shaped by both intrinsic genomic alterations and dynamic interactions with the tumor microenvironment (TME). Conventional analytical approaches offered limited insight into these complex mechanisms. However, the emergence of multi-omics technologies including genomics, transcriptomics, proteomics and spatial single-cell platforms dramatically expanded current understanding of tumor evolution, immune-stromal crosstalk and phenotypic plasticity. In the present review, findings from novel multi-omics studies were integrated to reframe PNET biology through the perspective of TME co-evolution. The present review highlighted how genomic instability serves as a key driver, promoting transcriptomic reprogramming and clonal evolution that subsequently remodels the TME into an immunosuppressive niche rich in cancer-associated fibroblasts and characterized by immune exclusion. The present review further emphasized the novel role of spatial multi-omics in deciphering the spatial heterogeneity of the PNET ecosystem. These insights accelerated the identification of novel biomarkers and revealed novel therapeutic susceptibilities, potentially paving the way for rational combination strategies that target both tumor-intrinsic pathways and microenvironmental constraints. Therefore, the present review proposed that multi-omics profiling provides not only a descriptive landscape but a mechanistic framework for precision oncology, enabling improved patient stratification and biomarker-driven therapeutic interventions in PNETs.

## 1. Introduction

Pancreatic neuroendocrine tumors (PNETs) are a rare (<2%) and biologically diverse class of neoplasms originating from the endocrine cells of the pancreas, distinguished by a complex pathological architecture and diverse clinical behavior (1–5). Despite a rare incidence (2.45 per 100,000) compared with pancreatic ductal adenocarcinoma, the 5-year survival rate for PNET is up to 40% (6,7). Particularly, PNETs exhibit a high propensity for liver metastasis and postoperative recurrence, with the liver being the predominant site of disease relapse (8–11). Based on hormone secretion, PNETs are broadly classified into functional and non-functional subtypes clinically. Functional PNETs (F-PNETs), such as insulinomas and gastrinomas, are typically diagnosed earlier due to hormone-related syndromes. By contrast, non-F-PNETs (NF-PNETs), which comprise the majority of cases, are often asymptomatic until late stages and indicate an extensive range of malignant potential. A recent study reported the 3.7-fold increasing incidence rate of NF-PNETs within the last 30 years (6,12,13). The World Health Organization (WHO) further stratifies PNETs into grades G1, G2 and G3 based on mitotic count and Ki-67 proliferation index (14,15). While G1 and G2 tumors are generally well-differentiated and may follow a more indolent course, G3 tumors encompass both well-differentiated high-grade PNETs and poorly differentiated pancreatic neuroendocrine carcinomas, each with distinct molecular and clinical behavior. Such evolutionary progression is generally indicative of poor clinical outcomes (16–19). Defining the evolutionary dynamics and progression characteristics of PNETs holds potential in enhancing prognostic assessment. Despite these established clinical frameworks, the management of PNETs faces challenges. A notable proportion of patients with well-differentiated tumors still experience relapse, underscoring the limitations of histopathological grading alone in predicting clinical trajectory. Furthermore, therapeutic options for advanced disease, including chemotherapy and targeted therapy (11,20,21), are limited by innate and acquired resistance mechanisms. This markedly highlights the unmet need for a more nuanced, molecularly driven understanding of PNET pathogenesis.

**Figure 1.**
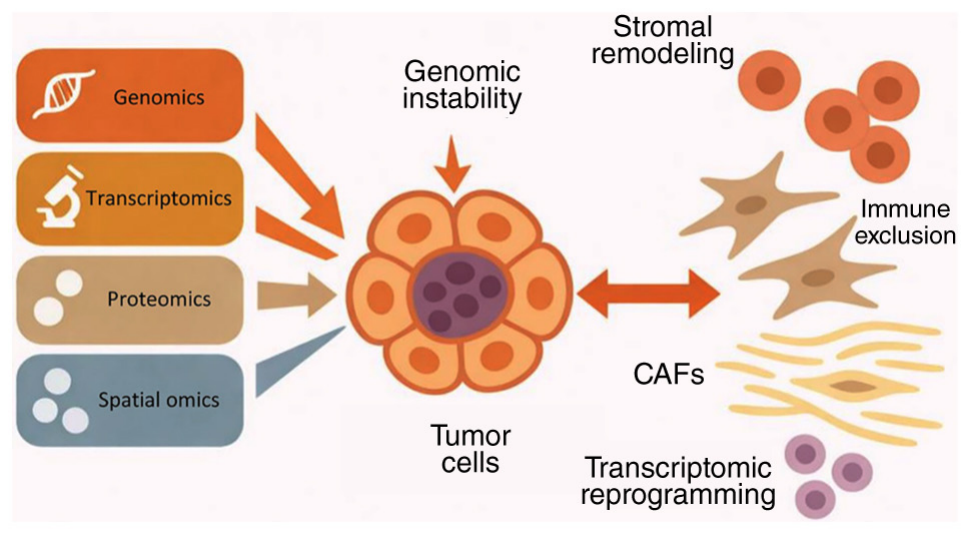
Multi-omics profiling of tumor progression and microenvironment in PNETs. Genomics, transcriptomics, proteomics and spatial omics collectively revealed how genomic instability in tumor cells is associated with transcriptomic reprogramming, CAF enrichment, stromal remodeling and immune exclusion in PNETs. Bidirectional interactions between tumor cells and the tumor microenvironment are illustrated to reflect the co-evolutionary dynamics. PNETs, pancreatic neuroendocrine tumors; CAF, cancer-associated fibroblast.

While the WHO grading system classifies tumors according to proliferative indices, it overlooks the functional plasticity and microenvironmental reprogramming that often underlie aggressive phenotypes in histologically similar tumors. Increasing evidence suggested that the tumor microenvironment (TME), a dynamic assembly of stromal cells, immune infiltrates, vasculature and extracellular matrix (ECM) serves a key role in regulating tumor progression, metastasis, therapeutic responsiveness and immune evasion (22–24). In several instances, TME features have exhibited stronger prognostic and predictive value compared with tumor-intrinsic factors alone, emphasizing the need to understand this intricate ecosystem (25–27). A key unmet need is the development of integrative approaches that place the TME at the forefront, alongside tumor-intrinsic factors, to improve prognostic accuracy and optimize therapeutic strategies in PNETs. Conventional siloed approaches, focusing exclusively on genomics or the immune compartment, have offered fragmented insights. Recent advances in multi-omics technologies including genomic sequencing, transcriptomic sequencing [bulk RNA sequencing (bulk RNA-seq), single-cell RNA sequencing (scRNA-seq) and spatial transcriptomics] and proteogenomic sequencing have enabled comprehensive profiling of both tumor-intrinsic and extrinsic features. In initial studies, genomic analyses revealed that multiple endocrine neoplasia type 1 (MEN1), death-associated protein 6 (DAXX) and α-thalassemia/mental retardation syndrome X-linked (ATRX) are among the most frequently mutated genes in PNETs, often associated with poor clinical outcomes and disrupted chromatin regulation (5,28–30). Beyond these earlier genomic profiling studies, the newer multi-omics platforms not only reconstruct the cellular architecture of PNETs but also reveal functional programs such as lineage plasticity, immune remodeling, metabolic reprogramming and tumor-stroma crosstalk that lead to disease progression. In the present review, findings from novel multi-omics studies were integrated to reframe current understanding of PNET biology through the lens of TME co-evolution (Fig. 1). The present review reveals core mechanisms such as genomic instability, stromal remodeling, immune modulation and biomarker identification, with a focus on the translational relevance for patient stratification and targeted therapeutic development.

## 2. Genomic landscape

### Genomic instability as a driver of dedifferentiation and transcriptomic plasticity

Notably, genomic and microenvironmental features vary markedly across the WHO tumor grades (G1, G2 and G3) as well as between functional and NF-PNETs, and findings from different studies should therefore be interpreted within the appropriate clinical and pathological context. In PNETs, genomic instability primarily refers to chromosomal instability characterized by copy number variations (CNVs), large-scale chromosomal gains and losses and telomere dysfunction associated with ATRX/DAXX alterations, rather than microsatellite instability or a high mutational burden (a threshold of >10 mutations/megabase), which are relatively uncommon in these tumors (5,28,29,31). Within this framework, genomic instability reflects progressive structural alterations of the genome that reshape transcriptional programs and cellular identity.

The transition from well-differentiated, slow-growing tumors (grade G1-G2) to poorly differentiated, high-grade PNETs is closely associated with increasing genomic instability and transcriptomic reprogramming. Cutting-edge technologies enabled the mapping of this transformation across multiple molecular layers (32,33). Low-grade tumors often present low CNV and maintain endocrine identity through the stable expression of lineage-specific transcription factors and chromatin regulators. However, as tumors progress to higher grades, extensive CNVs, chromosomal instability and altered DNA repair mechanisms emerge. These changes are accompanied by global shifts in gene expression, including downregulation of neuroendocrine markers and upregulation of genes associated with cell cycle progression, epithelial-mesenchymal transition (EMT), hypoxia and inflammation (34).

Single-cell and bulk transcriptomic analyses stratified by WHO grade (G1-3) revealed an association between tumor grade and increased proliferative signaling, loss of endocrine differentiation and activation of EMT and Myc-driven transcriptional programs in PNETs (35–37). CNV inference (inferCNV) from scRNA-seq in NF-PNETs demonstrated that G3 tumors harbor subclonal populations marked by chromosomal instability and dedifferentiation (38). These subclones preferentially localize to the invasive tumor margins and express markers of metastasis and cell cycle progression. Furthermore, in CNV-high tumors, extensive chromosomal aberrations, elevated tumor mutational burden (TMB) and activation of DNA repair and oxidative stress pathways are observed (34). These molecular features are often accompanied by microenvironmental alterations, including expansion of myofibroblastic cancer-associated fibroblasts (CAFs), ECM deposition and immune exclusion (39,40).

In addition to gene-level mutations, large-scale chromosomal alterations are a hallmark of PNET progression. CNV profiling using whole-genome sequencing revealed three major structural subtypes: CNV-low, -altered and -recurrent (38). CNV-low tumors typically retain endocrine differentiation and display stable karyotypes. By contrast, CNV-altered and -recurrent tumors exhibit broad chromosomal gains and losses. For example, recurrent CNV profiling by proteomic analysis identified frequent arm-level and focal gains and losses across the NF-PNET genome, even in tumors classified as low or intermediate grade (41). Common gains are observed on chromosomes 4p and q, 7p and q, 13q, 14q, 19p and q, 20p and q, while losses frequently affect 11p and q. Notably, amplification of CDK6 (chromosome 7) was functionally associated with enhanced cell cycle progression and defines a subset of highly proliferative tumors (42). Similarly, amplification of enhancer of zeste homolog 2, a histone methyltransferase also located on chromosome 7, may contribute to immune modulation by influencing interferon signaling and epigenetic silencing (43). By contrast, loss of MEN1 on chromosome 11, one of the most frequently deleted tumor suppressors in PNETs, disrupts chromatin regulation and is considered a fundamental early event in tumor initiation (28,30). Similarly, ATRX/DAXX mutations, often associated with alternative lengthening of telomeres, co-occur with chromosomal rearrangements involving chromosome 21q or 9p (44–46). The frequent alteration of MEN1, DAXX and ATRX genes underscores the notable importance of chromatin remodeling in PNET tumorigenesis. However, beyond the roles in initiation, these mutations appear to set the stage for later genomic catastrophes (5,31,47). The loss of function in these chromatin regulators likely creates a permissive state for the accumulation of CNVs and structural variations, ultimately promoting the transition from a well-differentiated to a poorly differentiated state (48). A notable case study described a patient with a germline Fanconi anemia group D2 protein (FANCD2) mutation whose PNET exhibited extensive chromosomal instability and a dedifferentiated transcriptional profile (49). Whole exome sequencing of this tumor revealed marked germline mutation of Wnt family member 10A (a Wnt signaling activator) and deletion of MutY homolog (a connection with base excision repair deficiency) (50–53). Furthermore, due to FANCD2 germline mutation, impaired DNA damage repair and unprotected genome resulted in high mutation rate in PNETs (54). Although increasing studies indicated high mutation frequencies (43%) (5,29), the prognostic impact of specific mutational co-occurrences remains less defined, suggesting context-dependent roles that warrant further investigation in larger, prospectively annotated cohorts (29,49).

Transcriptomic data including bulk RNA-seq, scRNA-seq, spatial transcriptomics and proteogenomic analysis further illuminate this process. scRNA-seq of primary tumors revealed enrichment of gene programs associated with angiogenesis and stemness (35). Particularly, transcription factor 4 (TCF4), which was reported to involve in neural development and neuroendocrine differentiation (55,56), enhanced activity in primary tumor sites. However, the markedly enriched cellular functions in metastatic tumor were associated with biosynthesis, mitosis, cell cycle and cell proliferation. Proteogenomic profiling revealed that high-grade tumors downregulate these markers and upregulate genes associated with hypoxia responses, EMT and an inflammatory signature, which associated with aggressive phenotype, such as invasion and metastasis (38,57). Spatial transcriptomics implied that these proliferative, dedifferentiated states are enriched at invasive and hypoxic regions with dense CAF infiltration and limited immune surveillance (44). In G3 PNETs, tumor cells and CAFs co-express EMT and invasion-associated genes (for example, MMPs), while CAFs secrete paracrine inducers of dedifferentiation, notably TGF-β1 (41,44,58,59). This reciprocal signaling loop may contribute to malignant transformation and illustrates the coupled evolution of tumor and stroma. In summary, these data supported that genomic instability appears to represent a key feature associated with tumor progression that reshapes cellular identity and primes tumors for invasion, metastasis and therapy resistance.

### Clonal evolution and the framework of metastatic niches

Longitudinal and cross-sectional integrated omics studies using pseudotime trajectory inference and CNV profiling has revealed dynamic clonal transitions during PNET progression. These transitions are marked by acquisition of metabolic plasticity, angiogenic signaling and transcriptional programs associated with immune evasion and proliferative expansion (35,48). These changes likely reflect selective pressures imposed by evolving microenvironmental niches. The cross-sectional snapshot provided by multi-omics data allows us to reconstruct these evolutionary trajectories, revealing the sequence of molecular events that culminate in metastatic competence.

Metastatic lesions, particularly in the liver, are often populated by tumor clones enriched in oxidative phosphorylation, Myc signaling and resistance to apoptosis depend on increased early region 2 binding factor family and Bax expression via scRNA-seq (35). These environments are also characterized by pro-angiogenic endothelial cells and M1-like macrophages, forming niches conducive to metastatic colonization. This metabolic phenotype may not only support the high energy demands of proliferation and invasion but also confer resistance to certain therapeutics (for example, targeted therapy). Spatial transcriptomics demonstrated that dissemination is often preceded by the emergence of spatially confined, proliferation-primed subclones at the tumor margin, frequently associated with hypoxia-driven gene expression (for example, MMP-9) (44). This finding suggested that metastasis may be preceded by transcriptional programs that facilitate dissemination orchestrated by the primary tumor.

Proteogenomic profiling delineated that EMT-like subpopulations, enriched in high-grade and metastatic tumors, indicate TGF-β activation, glycolysis, Myc signaling and hypoxia-responsive transcription (41,60,61). Particularly, hypoxia-induced EMT signature exhibits a positive association with TGF-β signaling pathway (57,62). scRNA transcriptomics identified that these metastatic clusters localize to invasive fronts with high CAF density and minimal T-cell infiltration (38). These findings suggested that metastatic competency may be preconfigured within transcriptionally primed subpopulations at the primary tumor margins. To define the metastatic potential in the high-grade tumors, scRNA transcriptome analysis in PNETs revealed the role of a gene signature with notable emergence of two genes proprotein convertase subtilisin/Kexin type 1 (PCSK1) and secreted modular calcium-binding protein 1 (SMOC1) (35,38). The emergence of PCSK1 and SMOC1 as potential markers of metastatic propensity is of notable translational interest. Validating these proteins as circulating biomarkers in patient plasma could potentially provide a non-invasive tool in assessing metastatic risk and guiding surveillance strategies for patients with localized disease in the future.

**Figure 2.**
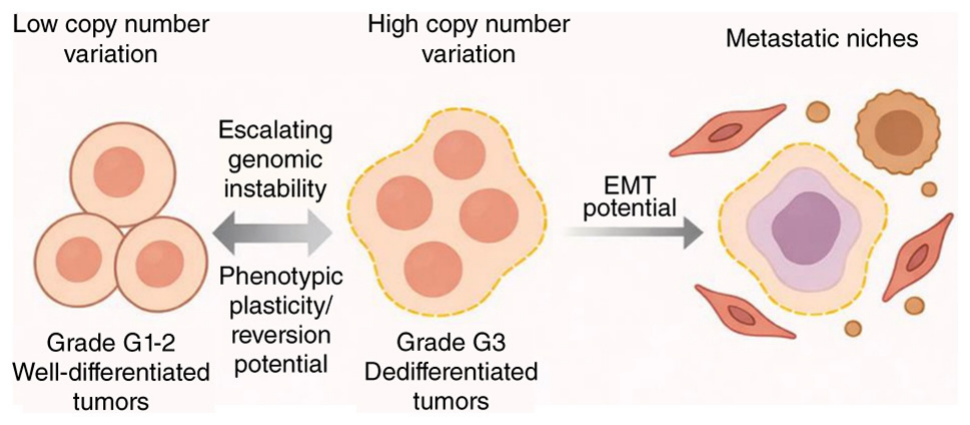
Genomic and evolutionary remodeling in PNETs. Schematic representation of how escalating genomic instability in PNETs promotes dedifferentiation and EMT. Low CNV tumors (grade G1-2) typically retain stable chromatin organization and endocrine identity. As CNVs and chromosomal aberrations accumulate, tumors acquire higher genomic complexity and progress to dedifferentiated states (grade G3). The bidirectional arrows indicate phenotypic plasticity and the potential for dynamic state transitions during tumor progression. These changes are reinforced by the TME. Multi-omics profiling has revealed these co-evolutionary dynamics between genomic alterations and the TME. EMT, epithelial-mesenchymal transition; CNV, copy number variation; PNETs, pancreatic neuroendocrine tumors; TME, tumor microenvironment.

In summary, genomic instability in PNETs promotes not only tumor-intrinsic transformation but also adaptation to selective pressures imposed by the microenvironment. The co-occurrence with dedifferentiation and stromal remodeling of genomic instability in PNETs underscores its key role in malignant progression (Fig. 2). These findings collectively illustrate the need for therapeutic strategies that account for intratumoral heterogeneity and spatial dynamics to effectively target the evolving landscape of PNETs.

## 3. Tumor-stromal-immune microenvironment

### Integrated omics deconstructing the PNET microenvironment

While tumor-intrinsic alterations are key to understanding PNET progression, a complete understanding requires dissecting the dynamic interactions of PNETs with the surrounding stroma and immune compartments. Traditional bulk sequencing approaches averaged signals across all cellular constituents, obscuring the unique contributions of rare but functionally key cell states. scRNA-seq and spatial transcriptomics revolutionized the ability to deconstruct the cellular heterogeneity of the PNET microenvironment. These high-resolution technologies exposed a complex ecosystem composed of malignant neuroendocrine cells, CAFs, endothelial cells and immune subsets including T and B cells, monocytic cell, tumor-associated macrophages and mast cells each exhibiting context-dependent differentiation states and functional programs (35,38). However, single-cell dissociation serves the key spatial context that defines cellular function. Spatial transcriptomic mapping demonstrated that these cellular populations exhibit non-random, spatially organized distributions (44,58). Proliferative tumor regions are typically bordered by dense fibroblast-rich stroma, which serves both as a physical barrier and a source of immunomodulatory cues. Proteogenomic profiling revealed that hypoxic and fibrotic zones often co-localize with reduced immune infiltration, highlighting spatial regulation of immune exclusion and therapeutic resistance (41). These technologies anchor the molecular profiles provided by scRNA-seq to a precise geographical location within the tumor architecture. These integrated omics approaches revealed that the spatial compartmentalization of the TME has notable clinical implications, which can explain the frequent failure of therapies that target ubiquitous molecules but fail to penetrate specific anatomical niches. Therefore, mapping the spatial TME may potentially provide a key foundation for the development of spatially informed combination therapies in the future.

### CAF-driven niches promote dedifferentiation and immune exclusion

Recent transcriptomic studies have identified distinct CAF subtypes in PNETs with specialized roles in shaping functional tumor niches (35,38,44). The functional diversification of CAFs appears to be closely associated with tumor grade progression (63–65). Spatial profiling of sorted α-smooth muscle actin^+^ stromal cells exhibited that CAFs evolve with tumor grade: G1/2 tumors exhibit ECM-remodeling CAFs (often termed myofibroblastic CAFs), while G3 tumors harbor immunoregulatory and pro-fibrotic CAFs (reminiscent of inflammatory CAFs) expressing TGF-β1, fibronectin 1 and fibroblast growth factor 8 (44,58). These changes contribute to immune exclusion, enhanced invasiveness and hypoxia (66). Furthermore, single-cell transcriptomic and spatial data reported that dedifferentiated tumor cells localize to CAF-enriched fibrotic zones, where EMT and cell cycle genes (for example, MMPs and Myc) are upregulated (38,44,58). These data illustrated how functional state transitions, such as the loss of neuroendocrine identity and acquisition of mesenchymal or progenitor-like traits, are not solely driven by intrinsic oncogenic signaling but are spatially reinforced by CAF-derived cues within the fibrotic tumor margins (58). This shift from a desmoplastic to an immunosuppressive CAF phenotype represents a key transition in PNET pathogenesis. Pseudotime trajectory analyses elucidated that proliferative, dedifferentiated subclones emerge along a continuum from endocrine to mesenchymal transcriptional states, particularly at hypoxic and fibrotic margins (35). Single-cell and proteomic profiling elucidated that tumor cells from patients with PNET within these micro-niches frequently express exhaustion markers (for example programmed cell death 1, lymphocyte-activation gene 3 and cytotoxic T-lymphocyte antigen 4), further reinforcing immune evasion (35,38,41,60).

Therefore, these observations underscored a model in which tumor cell state plasticity, stromal remodeling and immune exclusion co-evolve within spatially defined microenvironments. Dissecting these coupled networks at single-cell and spatial resolution is key to identifying actionable targets and designing therapies that disrupt both malignant progression and its supportive niche.

## 4. Immunological dimension

### Spectrum of immune engagement in PNETs

To fully understand the immunological consequences of stromal remodeling, the preset review focuses on the spatial and functional characterization of immune infiltration within the PNET microenvironment. The immune contexture of PNETs appears to be a key determinant of disease progression, metastatic potential and therapeutic responsiveness (67–69). Multi-omics approaches including bulk RNA-seq, scRNA-seq and immune deconvolution enabled the classification of PNETs into distinct immunophenotypes: Immune-cold, -active and -suppressive (41,60,70). According to low immune enrichment score, immune-cold tumors displayed sparse lymphocyte infiltration and diminished expression of antigen-presentation machinery. By contrast, the gene signature of the immune-hot tumor subgroup, defined by the nearest template prediction algorithm, demonstrated a notable association between activated inflammatory stromal response and immune suppression (60,71–73). Immune-active tumors, a subgroup of immune-hot tumor, are characterized by abundant cytotoxic T cells and M1-like macrophages, elevated interferon-γ signaling and upregulation of costimulatory molecules, features indicative of a partially activated or exhausted immune response (60,74). Immune-suppressive tumors with activated stroma display high infiltration of regulatory T cells, M2-polarized macrophages and elevated expression levels of immune checkpoint ligands such as programmed cell death-ligand 1 (75), indoleamine 2,3 dioxygenase 1 (IDO1) and T cell immunoglobulin and mucin-domain containing-3 (TIM-3), along with TGF-β pathway activation (41,60). Therefore, the immune-cold phenotype presents a unique clinical challenge; these tumors may be inherently less immunogenic due to low TMB or defects in antigen presentation machinery [for example, downregulation of major histocompatibility complex (MHC) class I], making them resistant to current immunotherapies (41). Furthermore, multiplex spatial profiles validated that these immune profiles are spatially compartmentalized within the TME of PNETs (70). The metastasis-like primary subtype defined by micro RNA profiles, presenting increased macrophages under hypoxia and necroptosis, represents a key axis of immune suppression (36,76). This creates a malignant cycle where hypoxia induces immunosuppression, which further promotes tumor growth and aggravates hypoxia. Although the clinical relevance of immunophenotypes cannot be overstated, these phenotypes are strongly associated with tumor grade and microenvironmental features, providing a key framework for prognostic stratification and identifies distinct patient subsets requiring tailored therapeutic approaches.

### Spatial and molecular determinants of immune modulation

The stromal barrier may serve a notable role in limiting immune infiltration (77). Spatially resolved transcriptomic and proteo-transcriptomic profiling of PNETs further clarified how architectural and molecular features of the TME regulate immune infiltration and function (44,60,78). Immune-active regions are often segregated by fibrotic stroma enriched in CAFs and immunosuppressive cytokines, which limit lymphocyte trafficking and activation. These barriers are maintained by signaling pathways such as TGF-β and Hippo-yes-associated protein (YAP)/transcriptional coactivator with PDZ-binding motif (TAZ), which promote ECM deposition and immune exclusion (59,61,79–81). By contrast, immune-permissive zones display higher expression levels of MHC class I and II molecules, chemokines and costimulatory ligands that facilitate effector T-cell recruitment and retention (41,82). The juxtaposition of immune-active and -silent regions within the same tumor reflected the spatial heterogeneity of immunological pressure and underscored the limitations of bulk profiling in capturing clinically relevant immune dynamics. Overall, these findings underscored the necessity of spatially integrated multi-omics approaches to fully resolve the immunological landscape of PNETs. The spatial compartmentalization of the immune response suggests that effective immunotherapy should overcome dual challenges: Boosting immune activation in permissive zones and breaking through immune suppression in excluded zones. This necessitates spatially informed combination strategies.

## 5. Biomarker identification and translational implications

The integration of multi-omics technologies accelerated the identification of clinically relevant biomarkers embedded within the PNET microenvironment. In addition to biomarker identification, the multi-omics studies discussed in the present review also highlight several molecular events and translationally relevant targets in PNETs, as summarized in Table I. These biomarkers may hold notable promise in refining prognosis, predicting therapeutic response and guiding personalized interventions, which can be categorized as prognostic, predictive and pharmacodynamic. Among the emerging candidates, versican (VCAN), a stromal proteoglycan enriched in mesenchymal subtypes was proposed as a prognostic biomarker and detectable in both tumor tissue and plasma of PNETs (60). For example, a patient with a localized G2 tumor but a VCAN-high/immune-cold profile might be considered for more frequent imaging or adjuvant therapy trials. Also, transcriptional co-activators YAP1 and TAZ, central mediators of Hippo pathway signaling, are preferentially activated in stromal-rich, immune-excluded tumors and represent potential therapeutic targets for patients with PNET (78). Predictive biomarkers are used to select patients for specific therapies. From an immunological perspective, the expression levels of immune checkpoint molecules including PD-L1, TIM-3 and IDO1 aligns with suppressive immune phenotypes and may identify patients most likely to benefit from immune checkpoint blockade (60). Furthermore, detecting specific mutations (for example, MEN1 loss) or activation signatures (for example, Myc and TGF-β) can guide the use of targeted agents, such as epigenetic modulators or kinase inhibitors. Lastly, pharmacodynamic biomarkers (for example, changes in circulating VCAN levels or immune cell subsets in peripheral blood) can be used in early-phase clinical trials to confirm that a drug is acting on its intended target and modulating the TME as expected (83,84).

Spatial and proteogenomic profiling further refines these associations by capturing context-specific expression patterns within distinct tumor-stroma compartments (58,60). These findings support a potential combination therapeutic strategy for stroma-rich PNETs. The proposed combination strategy (YAP/TAZ inhibition, immune checkpoint blockade and TGF-β suppression) warrants evaluation in rationally designed clinical trials, for example in patients with advanced, stroma-rich PNETs (78). The strategy emphasizes the value of multi-omics-informed stratification for personalized therapy. Furthermore, a previous case study of FANCD2 exemplified the potential of multi-omics for precision oncology; however, its extensive implementation faces notable hurdles (49). The high cost, complex data analysis and longer turnaround time of techniques like scRNA-seq currently preclude the use of multi-omics in routine diagnostics. The ultimate goal is a future where the treatment of each patient with PNET is guided by enhanced understanding of the unique ecosystem of the tumor, moving from a one-size-fits-all approach to personalized ecosystem-based therapy.

## 6. Artificial intelligence (AI) and computational integration in PNET research

The rapid expansion of multi-omics datasets in PNETs has generated novel opportunities in computational modeling and AI-driven identification. In recent years, machine learning and deep learning approaches have demonstrated growing utility in cancer diagnosis, grading and risk stratification across multiple tumor types, including breast and pancreatic cancer (85,86). In the context of PNETs, AI-assisted digital pathology and radiomics analyses may improve diagnostic accuracy, particularly in distinguishing well-differentiated G3 tumors from poorly differentiated neuroendocrine carcinomas, which often present overlapping morphological features (87–91). Image-based deep learning algorithms have the potential to integrate histological architecture with molecular signatures, thereby enhancing grading precision and predicting metastatic potential (88).

Beyond diagnostic applications, AI offers key tools in integrating complex multi-layered omics datasets. PNET biology is characterized by heterogeneous genomic alterations (for example, MEN1 and ATRX/DAXX mutations), metabolic reprogramming, stromal remodeling and immune heterogeneity (5,38,45). Computational frameworks such as graph-based neural networks, multi-modal data fusion algorithms and representation learning models may enable simultaneous integration of genomic, transcriptomic, proteomic and spatial transcriptomic data (92–95). These approaches could reveal latent molecular patterns that are not apparent through single-layer analysis and may improve the identification of clinically actionable subtypes. Notably, AI-driven modeling may facilitate prediction of TME interactions, vascular niche activation and potential immune evasion mechanisms (96).

Although AI applications in PNETs remain in early development and are not yet standardized for clinical practice, future directions are promising. Prospective integration of spatial transcriptomics with AI-based spatial modeling could enable reconstruction of tumor-stroma co-evolution dynamics (97,98). Furthermore, predictive modeling of therapeutic response-particularly to targeted inhibitors, anti-angiogenic agents or immune checkpoint blockade may improve patient stratification (97). However, challenges remain, including limited cohort sizes in rare tumors such as PNETs, data harmonization issues and the need for interpretability of AI models. Addressing these limitations through collaborative consortia and standardized pipelines will be key to translating AI-driven insights into precision oncology for patients with PNET.

## 7. Limitations and challenges of multi-omics approaches in PNET research

Despite the transformative potential of multi-omics technologies, several limitations should be considered when interpreting findings in PNETs. First, numerous multi-omics studies rely on relatively small patient cohorts due to the rarity of PNETs, which may limit statistical power and reduce the generalizability of findings (35,38,49). Second, technical variability arising from sample processing, sequencing platforms and data normalization strategies can introduce batch effects and complicate cross-study comparisons. In single-cell and spatial transcriptomic analyses, tissue dissociation procedures may alter cellular states or lead to selective loss of fragile cell populations, potentially biasing the representation of the TME (38,44,49). Furthermore, integrating heterogeneous data types, including genomics, transcriptomics, proteomics and spatial data remains computationally challenging and lacks standardized analytical pipelines (99). Lastly, while multi-omics studies have generated notable descriptive insights into TME interactions (35,58,78), translating these findings into clinically actionable biomarkers or therapeutic strategies requires further functional validation and prospective clinical studies. Addressing these challenges will be key to improving the reproducibility and clinical applicability of multi-omics research in PNETs in the future.

## 8. Future outlook and conclusions

**Figure 3.**
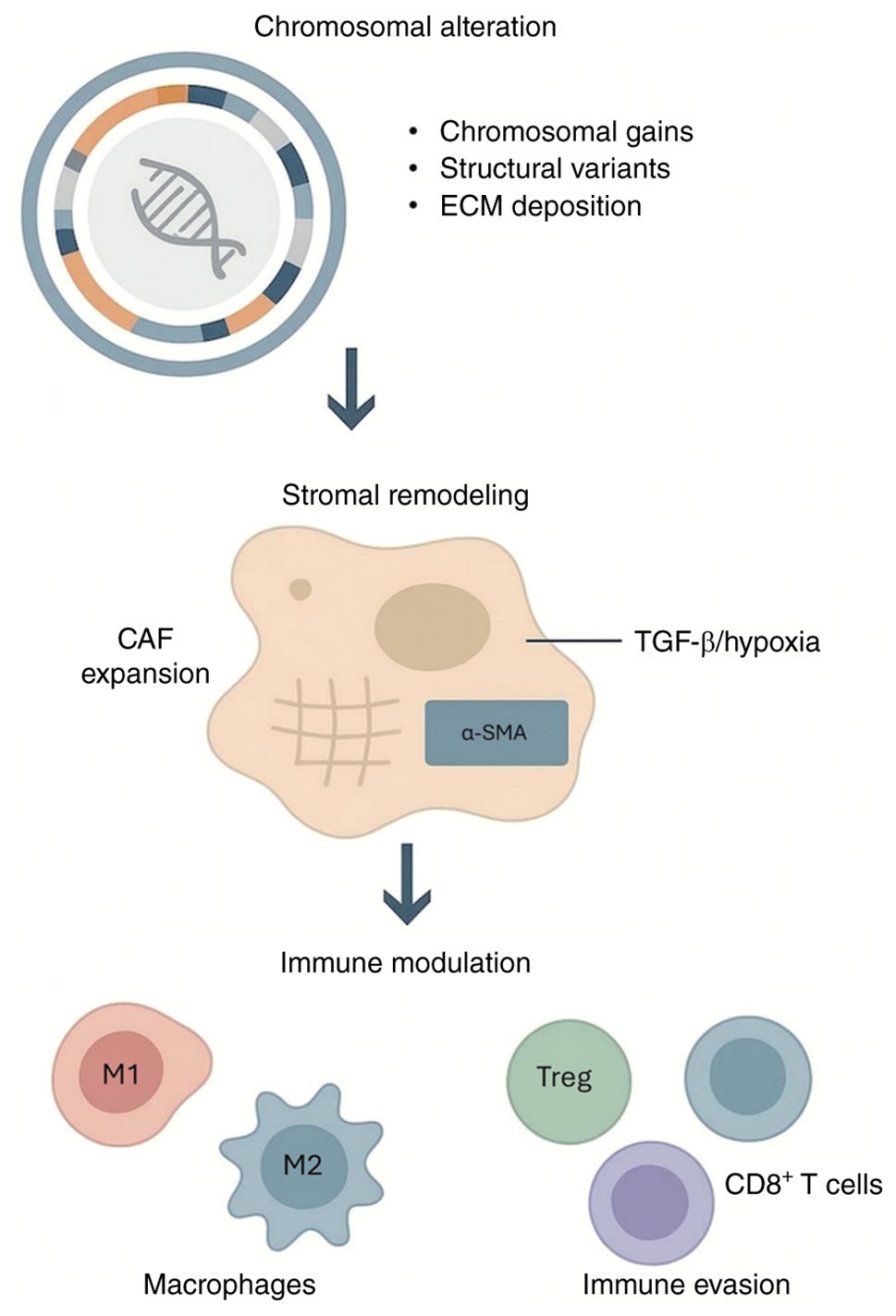
Chromosomal alterations orchestrate stromal remodeling and immune modulation in PNETs. Large-scale chromosomal gains, structural variants and ECM deposition contribute to stromal remodeling in PNETs. These stromal changes are associated with molecular drivers such as TGF-β signaling and hypoxia, which promote CAF activation and ECM remodeling. CAF expansion and activation, marked by α-SMA expression, contribute to ECM accumulation and fibrosis. The remodeled stroma shapes the immune landscape by polarizing macrophages (M1-like vs. M2), recruiting Treg cells and excluding CD8^+^ T cells, collectively establishing an immune-evasive microenvironment. ECM, extracellular matrix; CAF, cancer-associated fibroblast; PNETs, pancreatic neuroendocrine tumors; Treg, regulatory T cells; α-SMA, α-smooth muscle actin.

The advent of multi-omics technologies markedly transformed current understanding of PNETs. Once classified primarily by morphology and proliferation indices, these tumors are now recognized as dynamic, spatially structured ecosystems shaped by genomic instability, transcriptional diversity and microenvironmental remodeling (Fig. 3). The integrated evidence synthesized in the present review established that malignant progression in PNETs is not solely driven by cell-intrinsic mechanisms or extrinsic microenvironmental pressures alone, but by the continuous, bidirectional co-evolution of PNETs. Genomic instability may act as a key contributor to tumor evolution, not only promoting tumor cell dedifferentiation and heterogeneity but also actively remodeling the surrounding niche (38). By contrast, a microenvironment dominated by immunosuppressive CAFs and myeloid cells exerts potent selective pressures, fostering the expansion of subclones equipped with adaptive traits such as immune evasion and metabolic plasticity (66,100). This ‘genome-microenvironment’ co-evolution framework may provide a novel paradigm in understanding the aggressive and therapy-resistant nature of advanced PNETs.

Despite recent progress, several key questions remain unanswered, which may be explored in future research. The precise cell(s) of origin for different PNET subtypes remain to be elucidated. Furthermore, a key unanswered question is how therapeutic interventions (for example, targeted therapy and chemotherapy) reshape the spatiotemporal evolutionary trajectory of the PNET ecosystem. Prospective longitudinal studies incorporating multi-omics analysis of paired pre- and post-treatment samples (including liquid biopsies) will provide an enhanced view of resistance evolution. The metabolic coupling between tumor cells and stromal components is an underexplored area. Understanding how specific signals guide the formation of immune niches and how tumor-stroma metabolic interactions evolve under therapy is key to therapeutic reprogramming. Addressing spatial heterogeneity remains a major hurdle, as well as the identification of reliable biomarkers and the development of physiologically relevant preclinical models. Several strategies are warranted to progress research in this field. Advanced organoid and microfluidic systems will be key to modeling tumor-immune-stroma interactions. Humanized mouse models that preserve immune function will be key to validating novel therapies in the future.

In summary, validating the prognostic utility of multi-omics signatures warrants integration of multi-omics signatures into prospective clinical trials. For instance, a ‘window-of-opportunity’ trial design could be employed: Patients scheduled for resection of locally advanced PNETs receive a short course of a candidate agent (for example, a YAP/TAZ inhibitor or TGF-β blocker) prior to surgery. Paired pre- and post-treatment multi-omics analyses (scRNA-seq and spatial transcriptomics) of the resected specimens would then directly assess the impact of the drug on both tumor cell states and the TME, providing key mechanistic pharmacodynamic data. Furthermore, overcoming the technical and computational barriers to implementing these technologies in routine pathological workflows is a prerequisite for clinical translation. This will warrant the development of streamlined cost-effective assays and automated bioinformatics pipelines in the future.

## Figures and Tables

**Table I.** Key molecular events and translationally relevant targets discussed in PNETs.

Biological processes	Target/pathway	Study type	Mechanistic roles in PNETs	Potential translational implications	(Refs.)
Chromatin remodeling	MEN1	Genomic studies	Loss of MEN1 disrupts chromatin regulation and transcriptional control, contributing to tumor initiation	Molecular stratification; rationale for epigenetic susceptibility studies	(5,31,101)
Telomere maintenance/chromatin remodeling	ATRX/DAXX	Genomic studies	Mutations associated with ALT and chromosomal instability	Biomarker of aggressive biology; potential DNA repair or ALT-related susceptibility	(5,29,45)
Cell cycle regulation	CDK6	Proteogenomic analysis	Amplification promotes proliferation and cell cycle progression in aggressive tumors	Candidate target for cell-cycle-directed therapies	(42)
Epigenetic regulation	EZH2	Transcriptomic and proteomic studies	Histone methyltransferase involved in transcriptional silencing and immune modulation	Candidate target for epigenetic therapy	(43)
Stromal signaling	TGF-β1/TGF-β pathway	Spatial transcriptomics and proteomic studies	Promotes stromal remodeling, immune suppression and EMT-like phenotypes	Candidate target for stromal reprogramming and combination therapy	(41,44)
Hippo signaling	YAP1/TAZ	Multi-omics profiling	Activated in stromal-rich and immune-excluded tumors	Candidate target in stroma-rich PNETs	(78)
Immune evasion	PD-L1, TIM-3 and IDO1	Multi-omics profiling	Associated with immune-suppressive microenvironment and T-cell dysfunction	Biomarker-guided selection for immunotherapy-based approaches	(60)
Stromal biomarker	VCAN	Multi-omics biomarker studies	Enriched in mesenchymal/stromal subtype and detectable in tumor tissue and plasma	Prognostic biomarker and disease-monitoring candidate	
DNA damage response	FANCD2-associated DNA repair axis	Multi-omics biomarker studies	Stromal proteoglycan enriched in mesenchymal subtype; potential prognostic biomarker	Precision oncology rationale in selected genomically unstable cases	(49)

PNETs, pancreatic neuroendocrine tumors; FANCD2, Fanconi anemia group D2 protein; VCAN, versican; PD-L1, programmed cell death-ligand 1; IDO1, indoleamine 2,3 dioxygenase 1; TIM-3, T cell immunoglobulin and mucin-domain containing-3; YAP1, yes-associated protein 1; TAZ, transcriptional coactivator with PDZ-binding motif; EZH2, enhancer of zeste homolog 2; MEN1, multiple endocrine neoplasia type 1; DAXX, death-associated protein 6; ATRX, α-thalassemia/mental retardation syndrome X-linked; ALT, alternative lengthening of telomeres; EMT, epithelial-mesenchymal transition.

## Data Availability

Not applicable.
